# A Review of Sex Differences in Patients with Aortic Stenosis: A Focus on Diagnostic and Treatment Differences, a Narrative Review

**DOI:** 10.3390/jcm15166362

**Published:** 2026-08-18

**Authors:** Hilal Khan, Sophia Das, Rajiv Das

**Affiliations:** 1 Cardiothoracic Department, Freeman Hospital, Newcastle upon Tyne NE7 7DN, UK; 2 School of Medicine, University of Leeds, Leeds LS2 9JT, UK; 3 Faculty of Health and Life Sciences, Northumbria University, Newcastle upon Tyne NE1 8ST, UK

**Keywords:** aortic stenosis, sex differences, transcatheter aortic valve implantation

## Abstract

Severe aortic stenosis represents a growing burden of disease globally with a high risk of death if untreated. There are sex differences in the pathophysiological disease process affecting the aortic valve, patients’ clinical presentation, progression of valve disease, ventricular adaptation, timing of symptom onset, delays in diagnosis, and treatment strategies. Female patients are more likely than male patients to have preserved left ventricular function with concentric left ventricular hypertrophy and smaller left ventricular cavity sizes, which consequently produce low stroke volumes and more paradoxical low flow low gradient severe aortic stenosis. These differences can lead to diagnostic uncertainty resulting in delays to treatment and associated morbidity and mortality. The higher mortality rate reported in women in observational cohorts is multifactorial. It is influenced by delayed diagnosis, lower referral rates, older age at intervention and frailty; rather than female sex alone. There exist variations in the treatment and management of aortic stenosis with women being underdiagnosed and conservatively managed compared to men. A better understanding of the differences in pathophysiology, ventricular adaptation and haemodynamic effects between the sexes are key to improving diagnostic accuracy and referral for timely intervention. Women may require a more tailored approach to their lifetime management of aortic stenosis due to their smaller anatomy and greater comorbidity burden at presentation. This narrative review explores the epidemiology, pathophysiology, presentation, diagnosis and management of aortic stenosis in female patients.

## 1. Introduction

### 1.1. Overview of Aortic Stenosis

Aortic stenosis represents a growing burden of valvular heart disease (VHD) globally [1]. There are 158 cases of calcific aortic stenosis per 100,000 of the population with a two-fold increase seen between 1992 and 2021 [1]. Aortic valve disease is the commonest form of valve disease accounting for 61% of VHD [2]. Globally, rheumatic heart disease remains the commonest cause of aortic stenotic VHD, however in Europe and North America, calcific aortic stenosis is the commonest form of aortic stenotic VHD accounting for 43% of VHD [2,3]. Large contemporary registry data has shown significant 4-year mortality of 33.5% even with moderate asymptomatic aortic stenosis rising to 44.9% in patients with severe aortic stenosis irrespective of symptoms [4]. In patients with symptomatic severe aortic stenosis mortality has been shown to be as high as 90% at 5-years if left untreated, reflecting the severity of the condition and importance of prompt treatment [5].

### 1.2. Epidemiological Differences Between Men and Women in Aortic Stenosis

In the general population aortic valve calcification is more common in men than women, affecting approximately 10% of men between the ages of 50 and 65 years and 5% of women in the same age group. Aortic valve calcification increases with age irrespective of sex and at 80 years of age is present in approximately 37% of women and 55% of men, albeit the prevalence is lower among black men at 40% [6]. Risk factors such as hypertension, smoking and hyperlipidaemia are independently associated with an increased risk of aortic valve calcification [6,7]. Some studies however suggest that up to 60% of patients with severe aortic stenosis are women [8]. One reason for the lower numbers reported in other studies is the recognised delay in receiving a diagnosis of aortic stenosis for female patients, with only 64% of those with the condition receiving a diagnosis compared to 74% for men. This is a result of variations in female presentations with clinically significant aortic stenosis [9].

### 1.3. Pathophysiological Differences by Sex in Aortic Stenosis

There are key differences in the pathophysiological development of aortic stenosis in women compared to men. Women have less calcification and more fibrosis on their aortic valves compared to men [10]. Women can have up to 20% more dense fibrous connective tissue on their aortic valve compared to men presenting with aortic stenosis, thereby making identification of aortic stenosis more challenging in female patients [11]. This greater degree of fibrosis is the most likely explanation for women presenting with more symptoms at a later stage despite having less valvular calcification than male patients, as a significant proportion of the stenosis is mediated through fibrotic rather than calcific tissue in female patients [11].

The development of aortic stenosis is due to an interplay of mechanical stress, lipid accumulation and leaflet thickening. This is driven by different genes in men and women [12]. There is more downregulation of anti-inflammatory genes in men and upregulation of genes involved in calcification [12]. This process is attenuated in females partially by altered phosphorylation mechanisms which leads to reduction of the signalling involved in mineralisation of aortic valve tissue [13].

Furthermore, there are sex specific differences in the ventricular adaptation to aortic stenosis. Women present with more concentrically hypertrophied and less dilated ventricles whereas men present with eccentrically hypertrophied and more dilated ventricles and a reduction in left ventricular ejection fraction (LVEF). Concentric remodelling in women can lead to concentric left ventricular (LV) hypertrophy and diastolic dysfunction and has been found to be associated with an increased risk of all-cause mortality in women [14]. This may also explain the reason for the greater symptom burden observed in women despite lower degrees of aortic stenosis compared to men, due to chronic LV pressure overload being less well tolerated in hypertrophied smaller LV cavities with elevated LV filling pressures [14,15,16]. Insufficient oxygen supply to hypertrophied ventricles and impairment of myocardial perfusion reserve and fibrosis have been seen in women [10,17,18]. The genetic variations involved in driving these differences are not fully understood but may also be related to oestrogen mediated downregulation of the renin angiotensin system via downregulation of angiotensin 1, which likely explains the finding of more impaired LV function among men than women [15,19].

## 2. Methods

The literature search for this narrative review was conducted using PubMed and Google Scholar. The search terms used were (Gender Disparities in Aortic Stenosis), (Gender Disparities in TAVI), (Gender Disparities in Transcatheter Aortic Valve Replacement), (Gender Disparities in Transcatheter Aortic Valve Implantation), (Gender Disparities in SAVR), (Gender Disparities in Surgical Aortic Valve Replacement). All articles were screened after removing duplicates by 2 reviewers and studies were included if they reported all-cause mortality, provided data on aortic valve parameters and procedural complications. The search period was between January 2000 and August 2026 with a focus on articles published in the last 10 years.

## 3. Clinical Presentation and Imaging Variation Between the Sexes in Aortic Stenosis

**Figure 1:**
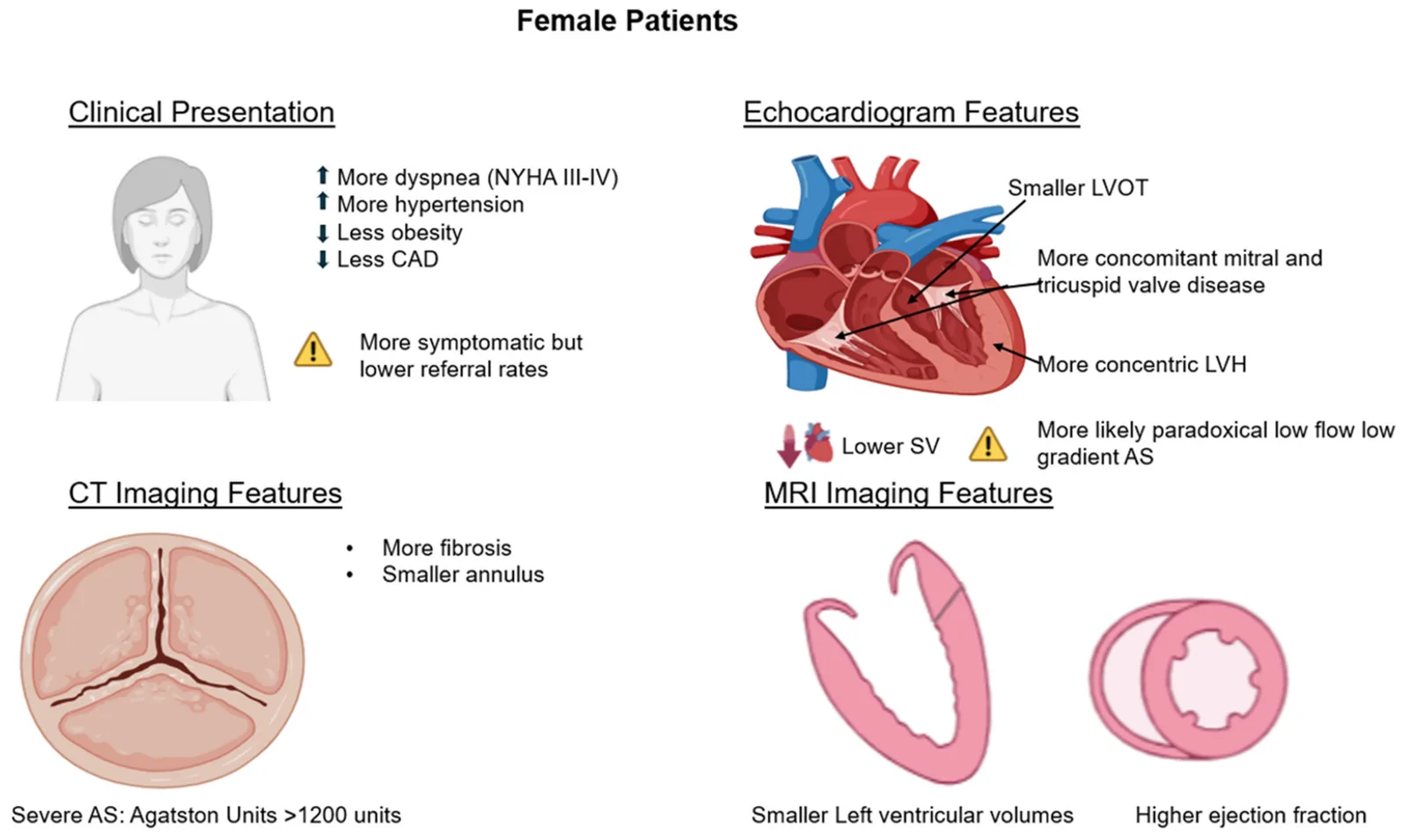
Differences in clinical presentation, echocardiographic, CT and cardiac MRI in female patients with severe aortic stenosis. AS, aortic stenosis; CAD, coronary artery disease; CT, computed tomography; LVH, left ventricular hypertrophy; LVOT, left ventricular outflow tract; MRI, magnetic resonance imaging; NYHA, New York Heart Association; SV, stroke volume.

There are significant variations in clinical presentation and imaging features between the sexes in severe aortic stenosis (Figure 1).

### 3.1. Clinical Presentation

Women with severe aortic stenosis can often be asymptomatic, leading to delays in diagnosis and referral for treatment [20]. When women do present, they are more likely to have more advanced New York Heart Association (NYHA) class III–IV dyspnoea compared to men [21]. Female patients with severe aortic stenosis are less likely to be obese than male patients with severe aortic stenosis [22]. Female patients with aortic stenosis are more likely to have hypertension but have less coronary artery disease than males with aortic stenosis [23]. Men are more likely to present with chest pain and a higher prevalence of coronary artery disease or peripheral vascular disease [24,25].

### 3.2. Echocardiographic Variation

There are considerable echocardiographic variations between the sexes in aortic stenosis. Women with severe aortic stenosis are more likely to have smaller LV outflow tracts, aortic roots and lower stroke volume velocities compared to male patients with severe aortic stenosis [26]. They are also more likely to have smaller LV cavity sizes with more concentric LV hypertrophy compared to male patients [25]. Female patients were more likely to have concomitant mitral and tricuspid VHD [25]. Men were more likely to have poorer LV function [25]. Women are more likely to present with paradoxical low flow low gradient severe aortic stenosis (pLFLG AS) which often leads to diagnostic uncertainty and underestimation of the severity of the stenosis which in turn may cause female patients to be undertreated [27]. pLFLG AS has a prognosis worse than normal-flow, high gradient severe aortic stenosis [28,29,30,31]. In the 2025 European Society of Cardiology and the European Association for Cardio-Thoracic Surgery (ESC/EACTS) guidelines, it is recommended that intervention should be considered in symptomatic patients with low flow (Svi < 35 mL/m^2^), low gradient (<40 mmHg) AS with normal LVEF (>50%) after careful confirmation that aortic stenosis is severe with a level of evidence IIa [32]. pLFLG AS is characterised by a reduced stroke volume (i.e., stroke volume index [SVi] ≤ 35 mL/m^2^), a reduced transvalvular mean gradient (<40 mmHg) and reduced transvalvular jet velocity (<4 m/s) despite severe orifice narrowing (aortic valve area ≤ 1 cm^2^) and a LVEF ≥ 50% [33,34]. Sex-specific thresholds of stroke volume index have been found to be independently associated with mortality using a cutoff 32 mL/m^2^ in women and 40 mL/m^2^ in men. Using the cut off levels of <32 mL/m^2^ for women and 40 mL/m^2^ for men, patients with pLFLG AS had the highest mortality in patients undergoing aortic valve replacement (AVR) [35,36]. Patients with pLFLG AS (LVEF ≥ 50%) are likely to require computer tomography (CT) imaging to assess the calcium burden on the valve to confirm the diagnosis of severity. Dobutamine stress echocardiography is of less value in female patients, who are more likely to have better LV function than men [37].

### 3.3. Computed Tomography Imaging Differences

The calcium burden on the aortic valve is a valuable marker of the severity of aortic stenosis. It has also been found to be a predictor of mortality in patients with aortic stenosis [38]. There are biological sex differences in the tissue composition of the aortic valve in patients with aortic stenosis. CT imaging of the aortic valve commonly demonstrates a greater degree of calcification in male patients compared to female patients [39]. In addition, for the same degree of aortic valve calcification women tend to experience more severe haemodynamic effects compared to men [40]. Female patients tend to present with a predominance of fibrosis on the aortic valve [41], which contributes to severe leaflet stiffening and valvular obstruction through the build-up of dense fibrotic tissue [39]. If the degree of calcification was used for prediction of aortic stenosis severity alone, without accounting for sex specific differences, there would be an underestimation of the severity of aortic stenosis among many female patients. This explains why a cut-off of 1200 Agatston units (AU) correlates with severe aortic stenosis in female patients compared to 2000 AU in male patients [32]. These thresholds are recommended by the ESC to guide decision making in classifying severe aortic stenosis [32]. The current method for classifying severe aortic stenosis by calcium burden does not account for valve size. A novel method which involves indexing aortic valve calcification to valve surface area has been suggested to improve the accuracy of aortic stenosis severity prediction [40].

Women with hemodynamically significant aortic stenosis, are found to have lower levels of calcium on the aortic valve than men [24,42,43]. A study found of 7864 patients with severe aortic stenosis undergoing AVR, found that women accounted for 80% of patients with small annuli (less than 21 mm) [44].

### 3.4. Cardiac Magnetic Resonance Imaging Differences

Women with severe aortic stenosis show smaller LV volumes on cardiac magnetic resonance (CMR) imaging compared to men, consistent with echocardiographic findings. Men were more likely to have lower LV ejection fraction as well as displaying more late gadolinium enhancement compared to women [15]. There is considerable discordance on CMR in remodelling patterns between men and women compared to echocardiographic features, however the significance of this is unclear [15].

## 4. Management and Outcome Variation Between the Sexes in Aortic Stenosis

### 4.1. Conservative Management

Female patients are frequently older at the time of presentation compared to male patients and more likely to have renal impairment [25]. They typically have higher surgical risk scores pre-operatively and are more likely to be treated conservatively compared to male patients [45]. This is partly driven by a greater proportion of women presenting with pLFLG AS and as a result being exposed to more watchful waiting approaches [21].

Interestingly, male patients, despite having poorer ejection fraction which is associated with poorer outcomes after transcatheter aortic valve implementation (TAVI), are more likely to undergo early TAVI [21]. Female patients are more likely to have pLFLG AS due to reduced stroke volume as a result of a smaller cavity size and are likely to do as well with TAVI as patients with classical high gradient aortic stenosis with preserved LV function, yet these patients are far less likely to undergo early TAVI [46]. Highlighting these discrepancies in care is key to improving current paradigms in delivering care across the female population [47].

Females are more likely to be symptomatic despite their lower rate of referral for treatment compared to males with symptomatic aortic stenosis [25]. The interpretation that lower rates of referral among women are entirely due to sex should be viewed with cautioun. The lower rate of referral for intervention is likely multifactorial. Women tend to present at an older age, with more comorbidities including renal dysfunction and greater frailty. These factors make treatment choice more complex and increase the risk of complications due to intervention. In addition, socioeconomic factors such as deprivation, healthcare access, language barriers and minority ethnicity may also contribute to lower referral rates among women. A large retrospective database study in England found that female gender, high deprivation and black or South Asian ethnicity were associated with a significantly reduced likelihood of receiving AVR [48].

### 4.2. Transcatheter Aortic Valve Implantation

Differences in men and women undergoing TAVI are shown in Table 1 [47,49,50,51,52,53,54,55,56]. Women undergoing TAVI are typically older than men and more likely to have higher mean aortic valve gradients at the time of TAVI [49]. They are also more likely to have smaller aortic valve annuli compared to men [53]. These features typically make them higher risk and less suitable for surgical aortic valve replacement (SAVR). Studies have shown better survival in women treated with TAVI compared to male patients [47].

The outcomes following TAVI compared to SAVR in women is an area requiring more research. The Randomised research in women all comers wIth Aortic stenosis (RHEIA) trial involving 443 women with severe aortic stenosis concluded that the incidence of death, stroke or rehospitalisation after 1 year was higher in SAVR than TAVI [57]. Among 1706 women involved in a meta-analysis at 1 year and 2 years TAVI patients had a significantly lower mortality than SAVR patients. In comparison there was no difference in mortality seen in males between TAVI and SAVR [58]. A post hoc analysis of the randomised Surgical Replacement and Transcatheter Aortic Valve Implantation (SURTAVI) trial found that, at 2 years, all-cause mortality or disabling stroke was similar between SAVR and TAVI for females and males at intermediate surgical risk [59,60]. A retrospective sub analysis of high-risk symptomatic patients with aortic stenosis from the Placement of Aortic Transcatheter Valve (PARTNER) trial concluded that female patients had lower late mortality rates in TAVI than SAVR [61]. The outcomes following TAVI by sex have been investigated in multiple trials. The PARTNER II S3 study investigated high and intermediate risk patients with severe aortic stenosis. The investigators concluded that there were no apparent sex specific differences in survival rates or rates of stroke in patients undergoing TAVI [49]. These findings were supported in the Cerebrovascular EveNts in patients undergoing TranscathetER aortic valve implantation with balloon-expandable valves versus self-expandable valves (CENTER) collaboration, which concluded that sex had no influence on 30-day mortality rate or stroke risk. However, the women in this trial had a higher incidence of life threating or major bleeding after TAVI [62].

The current ESC and American College of Cardiology/American Heart Association (ACC/AHA) guidelines recommend considering the patient age, life expectancy, anatomy, surgical risk, comorbidity, predicted durability, vascular access, patient preference and Heart Team Assessment when deciding whether TAVI or SAVR is most appropriate [32,63]. Although female sex alone is not an indication for TAVI it is an important factor to consider when making the decision between SAVR and TAVI. The female anatomy can influence risk of patient prosthesis mismatch and complexity of procedure in SAVR [40]. Moreover, women with aortic stenosis generally present at an older age, with more comorbidity and greater frailty than men which may influence the safety and outcomes of intervention [64]. Therefore, it is important to carefully consider all of the factors listed above when coming to a decision on what treatment option is most suitable for every patient with aortic stenosis.

Post TAVI female patients have comparable mean aortic valve gradients to male patients despite having smaller aortic annuli and aortic valve areas [56]. These features of TAVI in female patients compare favourably to SAVR in this group, with TAVI showing lower rates of patient prosthesis mismatch in women compared to SAVR with rates of up to 55% in some surgical series compared to 4.4% in TAVI studies [65,66]. As a result of their smaller annuli women also benefit from less paravalvular leak compared to men after TAVI [51]. There are different anatomical characteristics of the iliofemoral vessels, with women having smaller, calcified and more tortuous vessels [67]. Women are more likely to have vascular complications after TAVI compared to men, but this doesn’t seem to significantly affect mortality outcomes [54]. This is likely due to patient prosthesis mismatch having a greater impact on longer term outcomes after AVR and the ability of TAVI to mitigate this to a greater extent in women is likely responsible for the improved outcomes seen in women undergoing TAVI [68]. Despite studies demonstrating similar or better survival outcomes in TAVI compared to SAVR in female patients, there is an increased likelihood of vascular and access site complications such as bleeding and annular injury among females undergoing TAVI. In women, balloon expandable TAVI oversizing > 20% of the annular area was associated with an 8 times increased risk of annulus rupture [69,70]. Moreover, in an analysis of anatomical and procedural features associated with aortic root rupture, 74% of patients who experienced rupture during TAVI were women [69]. Older female patients are more likely to be anatomically predisposed to annular rupture [71]. An anatomical characteristic that predisposes a patient to annular rupture is a smaller aortic annulus [71]. A study involving 506 patients found that after correcting for body size and height women with severe AS had smaller aortic root dimensions, thus predisposing them to annular rupture [72].

### 4.3. Surgical Aortic Valve Replacement

Sex specific outcomes after SAVR are shown in Table 2 [73,74,75,76,77,78,79]. Women undergoing SAVR are at greater risk of patient prosthesis mismatch compared to men. This is associated with a significant increase in mortality ranging from 30–40% [80]. Studies have previously shown higher mortality in female patients undergoing SAVR compared to male patients [73]. This is almost certainly driven by the higher risk of patient prosthesis mismatch in women undergoing SAVR with up to 44% receiving a SAVR of ≤21 mm compared to only 2% in male patients [73]. There is not a considerable difference in long term valve durability seen in studies of SAVR and TAVI [81,82]. From a structural perspective TAVI is associated with greater paravalvular leak when compared to SAVR, however this is significantly less of an issue in female patients with smaller annuli who benefit from less paravalvular leak as well as the superior haemodynamics afforded especially by supra-annular TAVI valves [82]. However, treatment choice depends on age including life-time management, surgical risk, anatomy, valve durability, bicuspid valve status, coronary access, vascular access, frailty, and patient preference [32,42].

## 5. Discussion and Future Directions

### 5.1. Access to TAVI

**Figure 2:**
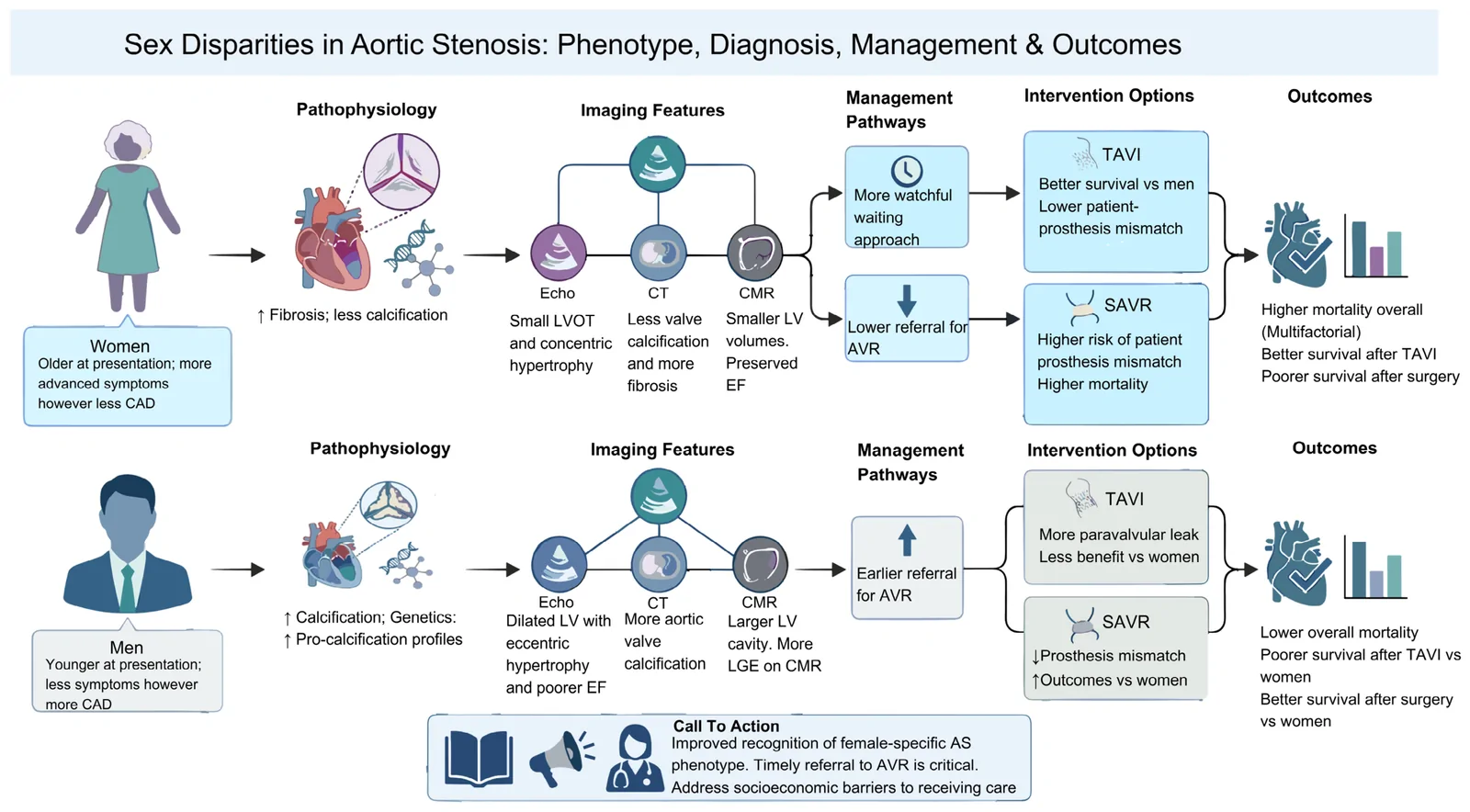
Central Illustration-Summarising sex disparities in aortic stenosis by phenotype, diagnosis, management and outcomes. AS, aortic stenosis; AVR, aortic valve replacement; CAD, coronary artery disease; CMR, cardiovascular magnetic resonance; CT, computed tomography; EF, ejection fraction; LGE, late gadolinium enhancement; LV, left ventricle; LVOT, left ventricular outflow tract; SAVR, surgical aortic valve replacement; TAVI, transcatheter aortic valve implementation.

Despite the favourable safety profile and mortality outcomes of women undergoing TAVI there are still significantly fewer women referred for TAVI compared to men in national audit data [83]. This is possibly due to greater rates of pLFLG AS in women which would benefit from similar treatment as classical high gradient aortic stenosis but may be treated conservatively for longer in female patients driving lower rates of referral for AVR, greater symptom burden at time of referral for AVR and conceivably contributing to poorer outcomes in female patients overall (Figure 2) [21,48].

### 5.2. Socioeconomic Influences on Accessing Care

Studies have also shown that women in more socioeconomically deprived neighbourhoods or from minority ethnic groups were also more likely to be significantly affected in their referral rates for AVR [48,84]. Careful attention especially to women from these groups is key to improving outcomes for women with severe aortic stenosis. These discrepancies are at least partially due to a lack of understanding of the variant presentations of cardiovascular disease more prevalent in women compared to men, resulting in fewer diagnostic tests and treatments being offered to women [85].

### 5.3. Prognosis

There is higher mortality overall in women with severe aortic stenosis compared to men, which is influenced by multiple factors including delayed diagnosis, lower referral rates for TAVI, older age at intervention and frailty compared to male patients [21]. There is however more favourable long-term survival in women undergoing TAVI compared to men [53]. This advantage is not seen in women undergoing SAVR when compared to male patients, possibly due to the higher rates of patient prosthesis mismatch in female patients which is associated with higher long-term mortality [68,73,80]. This paradox offers an opportunity to deliver care with improved longer-term outcomes in female patients with severe aortic stenosis.

### 5.4. Future Directions

Even with the increasing recognition and awareness of sex specific differences and gender related healthcare disparities in the presentation, diagnosis and treatment of aortic stenosis among women, there still exist gaps in knowledge from previous research. Historically women have been underrepresented in major valve trials resulting in limited evidence and uncertainty regarding management of this patient group (Table 3). Iribarren et al. reported that the number of women involved in relevant international trials was often below 50% of the study population and highlighted extrapolation of these results could be unreliable, therefore they recommended that more randomised clinical trials with a greater proportion of female participants are necessary [10]. A recent systematic review of 1079 cardiovascular clinical trials supported these findings and suggested the use of inclusive trial designs, better outreach strategies and regulatory policies to reduce inequality in female participation in clinical trials [86]. The Randomised researcH in womEn all comers with Aortic stenosis (RHEIA) trial is a good example of a randomised trial that has been used to inform management of women undergoing SAVR or TAVI [10,57]. There is limited evidence on the management of aortic stenosis in younger women, guidelines on the management of aortic stenosis have historically been based on older male populations [32]. This is an area that requires prioritisation for future research to improve patient outcomes in this group. Evidence shows that TAVI has beneficial outcomes in women, however the long-term durability of TAVI in women is currently an area with limited research [63]. Future research should address gaps in knowledge with randomised control trials powered for sex-specific analyses and more data is needed for durability of TAVI in women.

A greater focus on providing education to healthcare professionals regarding the variant clinical phenotypes present in women with all cardiovascular diseases including but not limited to aortic stenosis is key to improving health outcomes [85]. Future randomised controlled trials comparing outcomes after TAVI and SAVR in younger, lower risk female patients is needed with long-term follow-up to understand the impact of patient prosthesis mismatch and its longer-term effects on this patient cohort and whether it can be mitigated with TAVI valves with good long-term durability.

## 6. Conclusions

There are significant disparities seen between the sexes in the management of patients with aortic stenosis. One factor contributing to poorer outcomes seen in women is lower rates of referral for AVR compared to men. This is largely due to variations in the female phenotype of aortic stenosis which leads to more watchful waiting approaches. Women have a favourable response to TAVI with improved survival compared to men and better haemodynamic profiles compared to SAVR in women. However, treatment choice depends on age, surgical risk, anatomy, valve durability, bicuspid valve status, coronary access, vascular access, frailty, and patient preference. Improved education of female patterns of aortic stenosis and equitable access to AVR is essential to improve outcomes.

## Figures and Tables

**Table 1:** Studies summarising 1-year outcomes in women and men after transcatheter aortic valve implantation (TAVI). Data shows as mean ± SD or %. RCT = Randomised Controlled Trial; NR = Not reported.

Study	Numbers	Study Design	Statistical Adjustment	Age (Years)	Mean Aortic Pressure Gradient (mmHg)	Aortic Annulus Size (cm)	Vascular Complications	All-Cause Mortality at 1 Year	Paravalvular Leak	Mean Post Procedure Aortic Gradients (mmHg)	Mean Post Procedure Aortic Valve Area (cm^2^)
PARTNER 2 trial [49]	Women 657 Men 1004	RCT (post-hoc)	Adjustment for baseline covariates	82.5 ± 7.2 82.0 ± 7.1	47.8 ± 13.8 44.6 ± 12.8	2.1 ± 0.2 2.3 ± 0.2	7.2% 4.2%	9.4% 10.4%	4.2% 3.0%	12.89 10.43	1.49 1.81
Denegri et al. [50]	Women 2149 Men 1672	Observational	Propensity score matching	83 ± 6 81 ± 6	52.4 ± 15.3 47.3 ± 12.8	NR NR	13.7% 10.9%	11.5% 15.0%	10.3% 16.1%	8.0 ± 4.2 8.1 ± 4.1	NR NR
FRANCE 2 [51]	Women 1967 Men 2005	Observational	Adjustment for baseline covariates	84.0 ± 6.6 81.6 ± 7.5	51.0 ± 17.7 45.4 ± 14.8	NR NR	4.6% 1.9%	19.3% 23.7%	11.8% 17.1%	NR NR	1.1 1.0
POPULAR trial [52]	Women 466 Men 512	RCT	None	81.24 ± 5.46 79.39 ± 6.70	NR NR	NR NR	NR NR	8.2% 9.2%	NR NR	NR NR	NR NR
GALILEO [47]	Women 813 Men 831	RCT (post-hoc)	Adjustment for baseline covariates	81.12 ± 6.16 80.04 ± 6.93	NR NR	NR NR	NR NR	3.0% 6.1%	NR NR	9.9 ± 4.55 10.2 ± 4.73	1.76 1.94
PARTNER [53]	Women 1220 Men 1339	Observational	Adjustment for baseline covariates	84.9 ± 6.9 84.1 ± 7.3	46.1 ± 14.7 42.0 ± 13.7	1.83 ± 0.25 1.99 ± 0.27	17.3% 10.0%	6.48% 5.9%	6.0% 14.3%	9.76 8.91	1.57 1.83
Forrest et al. [54]	Women 1708 Men 1979	Observational	None	84.0 ± 7.6 82.7 ± 7.9	NR NR	NR NR	9.7% 4.9%	21.3% 24.1%	3.5% 4.8%	NR NR	NR NR
D’Ascenzo et al. [55]	Women 216 Men 161	Observational	Multivariable cox regression	82.9 ± 5.45 81.7 ± 5.32	56.4 ± 18.2 48.6 ± 13.9	NR NR	12.9% 9.8%	22.8% 30.8%	0.5% 1.3%	10.5 ± 6.6 10.3 ± 4.2	NR NR
Chandrasekar et al. [56]	Women 11,808 Men 11,844	Observational	Multivariable cox regression	82.28 ± 8.52 81.67 ± 8.63	NR NR	NR NR	8.27% 4.39%	21.3% 24.5%	3.1% 3.4%	5 6	1.8 2.0

**Table 2:** Studies summarising 1-year outcomes in women and men after surgical aortic valve replacement. Data shown as mean ± SD, median (interquartile range) or %. PSM = Propensity Score Matching; NR = Not reported.

Study	Numbers	Study Design	Statistical Adjustment	Age (Years)	Mean Aortic Valve Pressure Gradient (mmHg)	Aortic Valve Size (cm)	Major Bleeding	All-Cause Mortality at 1 Year
Andrei et al. [73]	Women 150 Men 478	Observational	PSM	60.7 ± 13.8 56.3 ± 13.6	43.4 ± 22.6 34.2 ± 21.3	44% ≤ 21 mm 2% ≤ 21 mm	NR NR	6.0% 3.2%
Chang et al. [74]	Women 18,720 Men 18,720	Observational	PSM and Bonferroni correction	64.4 ± 12.8 64.1 ± 12.8	NR NR	NR NR	NR NR	28.6% 31.2%
Fialka et al. [75]	Women 1272 Men 1272	Observational	PSM	67.3 ± 12.9 66.8 ± 13.4	NR NR	36.9% ≤ 21 mm 3.2% ≤ 21 mm	0.4% 0.4%	4.0% 4.0%
Myllykangas et al. [76]	Women 2814 Men 2814	Observational	PSM	72.2 ± 9.4 71.8 ± 9.1	NR NR	NR NR	1.4% 3.0%	7.1% 5.6%
Pawlik et al. [77]	Women 763 Men 763	Observational	PSM	67 (60–73) 67 (58–74)	NR NR	NR NR	NR NR	3.4% 3.5%
Zierer et al. [78]	Women 243 Men 433	Observational	PSM	59.8 ± 9.5 59.0 ± 9.7	46 ± 21 45 ± 18	43.2% ≤ 21 mm 7.4% ≤ 21 mm	11.9% 9.9%	1.8% at 2 years 1.4% at 2 years
Hernandez-Vaquero et al. [79]	Women 1618 Men 1618	Observational	PSM	59.9 ± 4.2 59.8 ± 4.1	NR NR	NR NR	NR NR	7.9% at 5 years 5.2% at 5 years

**Table 3:** Summarising the biological sex differences and gender-related healthcare disparities in women presenting with aortic stenosis.

Factor Influencing Diagnosis and Treatment of Aortic Stenosis in Women	Biological Sex Difference or Gender-Related Healthcare Disparity
Less aortic valve calcification	Biological sex difference
More aortic valve fibrosis	Biological sex difference
Smaller left ventricle size and left ventricular outflow tract	Biological sex difference
Concentric remodelling	Biological sex difference
More paradoxical low flow low gradient aortic stenosis	Biological sex difference
Smaller annular size	Biological sex difference
Lack of randomised control trials involving women and underrepresentation of women in previous trials.	Gender-related healthcare disparity
Lower referral rates for aortic valve replacement	Gender-related healthcare disparity
Most common clinical presentation dyspnoea as opposed to chest pain in men	Biological sex difference
Preserved ejection fraction	Biological sex difference
Reduced stroke volume	Biological sex difference
Healthcare access and socioeconomic status	Gender-related healthcare disparity
Risk of patient prosthesis mismatch	Biological sex difference

## Data Availability

No new data were created or analyzed in this study. Data sharing is not applicable to this article.
